# Isolated Cervical Seminoma Without Testicular or Mediastinal Primary: A Rare Extragonadal Germ Cell Tumor Presentation

**DOI:** 10.1155/crom/5148074

**Published:** 2026-09-23

**Authors:** Nikita Jhangiani, Saivaroon Gajagowni, Joanna Song, Gloria Mensah, Sergio Villegas De Leon, Michael Glover, Son Viet Nguyen

**Affiliations:** ^1^ Division of Internal Medicine, Baylor College of Medicine, Houston, Texas, USA, bcm.edu; ^2^ Department of Hospital Medicine, The University of Texas MD Anderson Cancer Center, Houston, Texas, USA, uth.edu; ^3^ Department of Genitourinary Medical Oncology, Division of Cancer Medicine, The University of Texas MD Anderson Cancer Center, Houston, Texas, USA, uth.edu

## Abstract

Germ cell tumors (GCTs) most commonly arise in the testes, with 1%–5% occurring at extragonadal sites, typically along the midline such as the mediastinum or retroperitoneum. We present a case of an exceptionally rare extragonadal seminoma confined to the cervical lymph nodes. Given the atypical location, diagnosis was difficult and required multiple biopsies to ensure appropriate correlation with histologic, immunophenotypic, and cytogenetic findings. Detection of 12p gain by FISH was pivotal in confirming germ cell origin. This case emphasizes the importance of maintaining a broad differential for atypical lymphadenopathy and will review the diagnostic challenge provided by this presentation.

## 1. Introduction

Germ cell tumors (GCTs) are the most common malignancies in young men and typically originate in the testes. A minority, approximately 1%–5%, arises in extragonadal locations, most often along the midline such as the mediastinum or retroperitoneum [1]. These extragonadal tumors are believed to develop from primordial germ cells that aberrantly migrate or fail to reach the gonadal ridge during embryogenesis. Cervical presentations of seminoma are exceedingly rare and can present a significant diagnostic challenge due to their nonspecific clinical and histopathologic features [2].

We report an unusual case of an extragonadal seminoma presenting as isolated cervical lymphadenopathy without evidence of a testicular or mediastinal primary. This case emphasizes the importance of keeping a broad differential for cervical lymphadenopathy. The atypical presentation presented a diagnostic challenge, requiring multiple biopsies and integration of immunohistochemistry (IHC) and genetic studies.

## 2. Case Presentation

### 2.1. Patient Background

Our patient is a 34‐year‐old male with no significant medical history and only medication use being an antihistamine for seasonal allergies. He is a nonsmoker, and family history is notable for multiple members with head and neck cancer secondary to smoking. He presented with 1 month of pain and increasing swelling of a right neck mass below his earlobe. He denied fever, chills, night sweats, weight loss, dysphagia, odynophagia, cough, or respiratory symptoms. Initial contrast‐enhanced CT of the neck demonstrated three heterogeneous enhancing right Level II lymph nodes with central low attenuation, suspicious for necrotic nodes. The largest measured 1.2 × 1.9 × 2.8 cm. No pharyngeal mass was identified.

### 2.2. Initial Diagnostic Evaluation

An ultrasound‐guided core needle biopsy of the largest right cervical lymph node revealed a metastatic poorly differentiated malignant tumor. Histology showed sheets and nests of tumor cells with pale to clear cytoplasm and high‐grade nuclei with prominent nucleoli, interspersed with nonnecrotic granulomas. IHC demonstrated tumor cells negative for CK7, CK20, p16, S100, CDX2, TTF‐1, NKX3.1, PAX8, CK5/6, p63, CD34, cytokeratin AE1/AE3, CD45, CD56, EMA, SOX10, melanoma cocktail, MART‐1, and HMB45. HNF1*β*, FOXA2, glypican‐3, or GATA3 were not tested. SALL4 staining was positive on outside review, suggesting GCT origin. Flow cytometry was negative for monoclonal B‐cell or aberrant T‐cell populations, excluding lymphoma.

Serum tumor markers, including LDH (173 U/L), AFP (< 2 IU/mL), and *β*‐hCG (< 1 mIU/mL), were normal. Scrotal ultrasound was completely unremarkable, with no mass lesions identified. There were no scars, calcifications, atrophy, heterogeneity, or hypoechogenicity to suggest a burnt‐out primary testicular tumor. PET‐CT revealed mildly to moderately enlarged, markedly FDG‐avid right jugular nodes and a single left supraclavicular node, consistent with metastatic disease. A mildly FDG‐avid subcentimeter left hilar node (maximum uptake of 4.5 SUV) and right posterior mediastinal node (maximum uptake of 4.48 SUV) were also noted. These nodes were reported to potentially represent metastatic or reactive changes.

### 2.3. Hospital Presentation

**Figure 1:**
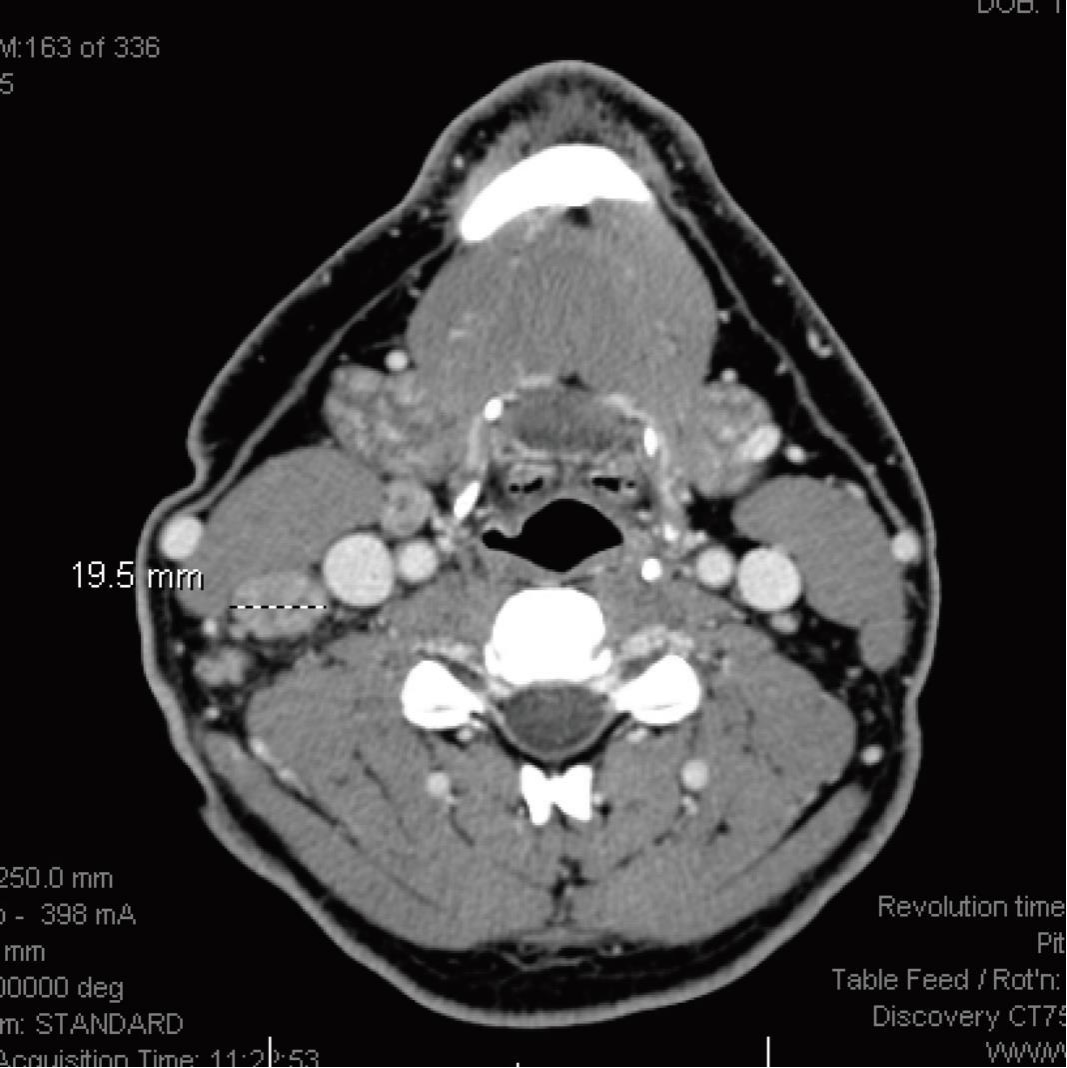
CT soft tissue neck showing the largest lymph node measured in the coronal plane [3].

The patient presented to our hospital for cancer‐related pain and a second opinion after being advised at an outside hospital to undergo bilateral orchiectomy empirically, despite a negative testicular ultrasound and PET‐CT. He again reported a rapidly enlarging, painful right neck mass without systemic symptoms. Repeat testicular ultrasound was performed at our institution, which again identified no suspicious lesions, scars, calcifications, or changes in echogenicity. Contrast‐enhanced CT of the chest demonstrated no mediastinal mass or intrathoracic malignancy. Mildly prominent multicompartmental mediastinal and bilateral hilar nodes were noted; however, they were measured no greater than 10–11 mm. These nodes were interpreted as nonspecific or reactive. CT of the neck demonstrated enlarged, morphologically suspicious lymph nodes in the right neck at Levels IIA, IIB, and III, the largest node now 1.6 × 2.0 × 3.0 cm (Figure 1).

After multidisciplinary discussion with oncology and pathology, a liquid biopsy, repeat in‐house fine‐needle aspiration (FNA) and core needle biopsy, and direct laryngoscopy were recommended due to the atypical presentation, incomplete molecular diagnostics, and family history. Direct laryngoscopy was negative for primary lesions. The liquid biopsy identified multiple single‐nucleotide variants involving ASPM, CHD2, FH, KRAS, PIK3CD, and TP53, all detected at very low variant allele frequencies (< 1%). No high‐level clonal or dominant genomic alterations were identified. FNA with core biopsy showed sheets of malignant cells with clear cytoplasm and prominent nucleoli. IHC was positive for SALL4, OCT3/4, and SOX17 and negative for cytokeratin cocktail, PLAP, EMA, CD117, CD30, CD3, and CD20, favoring a diagnosis of seminoma. Given extensive necrosis and crush artifact, further pathologic evaluation could not be completed. A repeat biopsy was performed to obtain tissue for FISH for iso(12p). Two subpopulations of cells demonstrated 4–7 copies of 12p13.3 relative to 12cen in 11%–24% of cells, consistent with 12p gain, supporting GCT cytogenetics.

### 2.4. Outcome

Repeat staging CT of the neck, chest, abdomen, and pelvis showed stable to slightly enlarged right cervical adenopathy, the largest node now 1.9 × 2.2 × 3.2 cm, unchanged small‐volume mediastinal lymphadenopathy, and no pathologically enlarged abdominal nodes. Both testicles were normal. Pulmonary function testing was normal.

The tumor was classified as a Stage IIIB GCT (seminoma) with an atypical presentation confined to cervical lymph nodes and no detectable mediastinal or testicular primary. The mediastinal lymphadenopathy was deemed to be reactive/insignificant given its posterior location and unchanged appearance, with a note to continue monitoring in future imaging studies. The patient was admitted for initiation of systemic therapy with bleomycin, etoposide, and cisplatin (BEP) chemotherapy, and no orchiectomy was recommended.

Following the first cycle of BEP, the patient developed a non‐ST‐elevation myocardial infarction requiring percutaneous coronary intervention with placement of a drug‐eluting stent in the left anterior descending coronary artery. Bleomycin was subsequently discontinued, and he completed a total of four cycles of etoposide and cisplatin (EP).

Interval CT imaging after treatment demonstrated a marked reduction in the cervical lymphadenopathy, with the dominant right Level II node decreasing from approximately 2.4–1.2 cm. Mediastinal and hilar lymph nodes remained stable without evidence of progression. End‐of‐treatment FDG‐PET/CT demonstrated no evidence of FDG‐avid malignancy. A small residual 1.1‐cm right Level IIB lymph node showed no abnormal FDG uptake and was interpreted as treated residual disease. Previously FDG‐avid cervical and supraclavicular lymph nodes had completely resolved. The patient remains on surveillance with no evidence of recurrent disease at his most recent follow‐up.

## 3. Discussion

GCTs are the most common malignancies in young men and typically arise within the testes. Approximately 1%–5% originate in extragonadal sites, most often along the midline—such as the anterior mediastinum or retroperitoneum—reflecting aberrant retention of primordial germ cells during embryonic migration [1]. Recent systematic review data further demonstrate that GCT pathology can occur at aberrant sites, including cervical lymph nodes, reinforcing the importance of maintaining a broad differential diagnosis for atypical cervical lymphadenopathy [4]. Lateralized extragonadal presentations, particularly in the cervical lymph nodes, are exceptionally rare and diagnostically challenging.

This patient presented with isolated right cervical lymphadenopathy and no evidence of testicular or anterior mediastinal disease on serial imaging, suggesting a true extragonadal origin. The absence of a detectable primary lesion distinguishes this case from the more common pattern of metastatic seminoma with occult or “burned‐out” testicular disease, reported in up to 12% of GCTs [2]. Such atypical presentations often lead to a broad differential diagnosis, including lymphoma, metastatic squamous cell carcinoma, and melanoma.

Guidelines from the American Academy of Otolaryngology–Head and Neck Surgery recommend that any adult neck mass be considered malignant until proven otherwise [5]. Our patient′s lymphadenopathy was concerning for malignancy given the persistence (> 2 weeks), firmness, and size > 1.5 cm. Evaluation should include imaging, laboratory testing, and FNA with core needle biopsy. FNA uses a thin needle (typically 22–25 gauge) to aspirate individual cells or small cell clusters for cytologic examination [6]. Core needle biopsy uses a larger hollow needle (typically 14–18 gauge) to obtain cylindrical cores of tissue. Unlike FNA, core needle biopsy preserves tissue architecture, allowing histologic evaluation, IHC, and molecular studies (e.g., FISH) [6]. Excisional or open biopsy is a surgical procedure in which the entire lymph node or lesion is removed for complete histopathologic examination. While it provides the greatest amount of tissue, it is not recommended as the initial diagnostic procedure for most adult neck masses because it may delay definitive treatment and increase the risk of tumor seeding or compromise subsequent surgical management [5]. Laryngoscopy is also critical, as most cervical lymphadenopathy represents metastases from head and neck primaries [7].

Cytogenic, histologic, and immunophenotypic evaluation played a decisive role in this diagnosis. The liquid biopsy identified missense variants across several genes, none typical of GCTs and all detected at very low variant allele frequencies (0.5%–1%). At these levels, the findings most likely reflect small DNA subpopulations rather than dominant or driving tumor clones. Seminomatous cells typically exhibit clear cytoplasm, distinct nucleoli, and a lymphoid stroma, with strong nuclear expression of OCT3/4 and SALL4 and, variably, SOX17 [2]. The tumor in this case showed this characteristic profile, while lacking cytokeratin expression (AE1/AE3), epithelial markers, and lineage‐specific antigens, effectively excluding carcinoma, lymphoma, and melanoma. Although histologic interpretation was complicated by crush artifact and necrosis (a well‐recognized pitfall in small biopsies) [2], cytogenetic testing provided confirmatory evidence.

Detection of 12p amplification by FISH supported the diagnosis, as isochromosome 12p or 12p gain remains the cytogenetic hallmark of GCTs and is particularly valuable in histologically ambiguous or extragonadal cases [2]. However, emerging studies have challenged a purely germ‐cell lineage model for all germ cell neoplasms [8]. Recent genomic analyses, particularly in yolk sac tumors and somatic‐type malignancies arising within GCTs, have suggested that some tumors may develop through mechanisms of somatic transformation, lineage plasticity, or transdifferentiation [8]. However, these findings have primarily been described in nonseminomatous GCT components and should not be directly extrapolated to pure seminoma. Seminoma continues to be considered a tumor of primordial germ cell origin, with characteristic morphology, immunophenotype, and recurrent molecular alterations including chromosome 12p gain [9]. In our patient, the combination of classic seminoma histology, absence of a gonadal primary, and demonstration of 12p gain supports the diagnosis of primary extragonadal seminoma.

Management of extragonadal seminoma parallels that of primary testicular seminoma. Platinum‐based combination chemotherapy with BEP remains the standard of care, achieving cure rates exceeding 80% even in advanced disease [2]. The patient′s classification as Stage IIIB disease and initiation of BEP chemotherapy align with established protocols. Surgical resection or radiation is generally reserved for residual or refractory disease after systemic therapy.

Radical inguinal orchiectomy is typically the first diagnostic and therapeutic step in suspected testicular GCTs because the testis is the most common primary site, and orchiectomy provides definitive tissue for histologic confirmation, staging, and risk stratification [10, 11]. Empiric orchiectomy may be considered when imaging strongly supports an occult testicular primary, as “burnt‐out” testicular tumors can present with metastatic disease despite minimal or absent sonographic findings [12]. However, guideline‐based management requires evidence of a testicular primary—either a palpable mass, intratesticular lesion on ultrasound, or pathologic confirmation—before proceeding with orchiectomy.

In this patient, both testicular ultrasound and PET‐CT demonstrated no evidence of a testicular mass, and two biopsies of the cervical lymph node failed to show malignancy consistent with a primary gonadal origin. Thus, recommending bilateral orchiectomy at the outside hospital was inconsistent with established testicular cancer guidelines, as there was no radiologic or histologic proof that the malignancy originated in the testes.

Response assessment following chemotherapy for seminoma differs from that of nonseminomatous GCTs. Current EAU and NCCN guidelines recommend FDG‐PET/CT no sooner than 6 weeks after completion of chemotherapy to minimize false‐positive findings related to posttreatment inflammation [10, 11]. Residual masses < 3 cm generally require surveillance alone, whereas PET/CT is recommended for residual masses > 3 cm to distinguish viable tumor from fibrosis or necrosis [11]. In this patient, posttreatment PET/CT performed after completion of chemotherapy demonstrated no FDG‐avid disease, with only a small nonavid residual cervical lymph node, supporting surveillance without additional intervention.

Patients with extragonadal seminoma generally achieve outcomes comparable to those with primary testicular seminoma when treated with platinum‐based chemotherapy. If relapse occurs following first‐line treatment, salvage chemotherapy with TIP (paclitaxel, ifosfamide, and cisplatin), VIP (etoposide, ifosfamide, and cisplatin), or high‐dose chemotherapy with autologous stem cell transplantation may be considered in appropriate candidates according to current guideline recommendations [10, 11].

This case adds to the limited body of literature describing seminoma manifesting as isolated cervical lymphadenopathy without a definite midline or testicular primary. It underscores the value of comprehensive immunohistochemical and molecular evaluation in resolving diagnostic uncertainty and demonstrates the utility of 12p FISH testing as a confirmatory adjunct. Recognition of this unusual presentation expands the clinical spectrum of extragonadal GCTs and reinforces the importance of molecular confirmation in establishing the diagnosis.

## Funding

No funding was received for this manuscript.

## Consent

The patient provided verbal consent to publish this case report. The authors have removed all direct and indirect patient identifiers, and the report has been sufficiently anonymized in accordance with the ICMJE Recommendations such that the risk of patient identification is minimized.

## Conflicts of Interest

The authors declare no conflicts of interest.

## Data Availability

Data sharing is not applicable to this article as no datasets were generated or analyzed during the current study.

## References

[1] Bokemeyer C. , Extragonadal Germ Cell Tumors: Clinical Presentation and Management, Seminars in Oncology. (2007) 34, no. 3, 250–259, 17911410.

[2] Bhardwaj S. , Salimian M. , Matoso A. , Gross J. , Argani P. , and Baraban E. , Establishing the Diagnosis of Germ Cell Tumors in Patients Presenting With Metastatic Disease, American Journal of Surgical Pathology. (2025) 49, no. 11, 1097–1104, 10.1097/PAS.0000000000002438, 40495550. 40495550

[3] Nguyen S. , Computed Tomography of Neck, 2025, The University of Texas MD Anderson Cancer Center.

[4] Costa Silva A. , Morgado A. , Alturas Silva J. , Oliveira P. , Clarke N. , Almeida Pinto R. , and Gulamhusein A. , Retroperitoneal Lymph Node Dissection for Growing Teratoma Syndrome in Testicular Cancer: A Systematic Review of Surgical Outcomes, World Journal of Urology. (2026) 44, no. 1, 10.1007/s00345-026-06207-5, 41528533. PMC12799728 41528533

[5] Pynnonen M. A. , Gillespie M. B. , Roman B. , Rosenfeld R. M. , Tunkel D. E. , Bontempo L. , Brook I. , Chick D. A. , Colandrea M. , Finestone S. A. , Fowler J. C. , Griffith C. C. , Henson Z. , Levine C. , Mehta V. , Salama A. , Scharpf J. , Shatzkes D. R. , Stern W. B. , Youngerman J. S. , and Corrigan M. D. , Clinical Practice Guideline: Evaluation of the Neck Mass in Adults, Otolaryngology–Head and Neck Surgery. (2017) 157, no. S2, S1–S30, 10.1177/0194599817722550, 28891406. 28891406

[6] Stewart C. J. , Coldewey J. , and Stewart I. S. , Comparison of Fine Needle Aspiration Cytology and Needle Core Biopsy in the Diagnosis of Radiologically Detected Abdominal Lesions, Journal of Clinical Pathology. (2002) 55, no. 2, 93–97, 10.1136/jcp.55.2.93, 11865001. PMC1769583 11865001

[7] Calabrese L. , Jereczek-Fossa B. A. , Jassem J. , Rocca A. , Bruschini R. , Orecchia R. , and Chiesa F. , Diagnosis and Management of Neck Metastases From an Unknown Primary, Acta Otorhinolaryngologica Italica. (2005) 25, no. 1, 2–12, 16080309. PMC2639847 16080309

[8] Ricci C. , De Leo A. , Grillini M. , Orsatti A. , Lobo J. , Fernanda-Pontes F. , Tavares N. T. , Vieira J. , Ambrosi F. , Grillini A. , Franchini E. , Vasuri F. , Nosseir S. , Mollica V. , Massari F. , Acosta A. M. , Fiorentino M. , Li H. , and Baraban E. , Immunohistochemical Characterization of Somatically Derived "Yolk Sac Tumors": Analysis of a Multi-Institutional Case Series, Virchows Archiv. (2026) 474, 1–6, 10.1007/s00428-026-04622-y, 42313184. 42313184

[9] Lobo J. , Tavares N. T. , Fernandes-Pontes F. , Fonseca D. , Jerónimo C. , Henrique R. , Ricci C. , de Leo A. , Collins K. , Idrees M. T. , Ulbright T. M. , Gupta S. , and Acosta A. M. , Phenotypic and Molecular Features of Somatically Derived "Yolk Sac Tumors": Similarities and Differences With Counterparts of Germ Cell Origin, Modern Pathology. (2025) 38, no. 9, 100807, 10.1016/j.modpat.2025.100807, 40484319. 40484319

[10] Gilligan T. , Lin D. W. , Adra N. , Bagrodia A. , Feldman D. R. , Yamoah K. , Aggarwal R. , Chandrasekar T. , Costa D. , Drakaki A. , Eggener S. , Emamekhoo H. , Geynisman D. M. , Graham L. , Humphrey P. , Leuva H. , Levine E. G. , Luckenbaugh A. , Maughan B. L. , McGregor B. , Monk P. , Picus J. , Pierorazio P. , Rais-Bahrami S. , Reichert Z. , Rwigema J. C. , Saylor P. , Shah A. , Shah S. , Singla N. , Sircar K. , VanderWeele D. , Zhumkhawala A. , Montgomery S. , and Sliker B. , NCCN Guidelines Insights: Testicular Cancer, Version 2.2025, Journal of the National Comprehensive Cancer Network. (2025) 23, no. 4, e250018, 10.6004/jnccn.2025.0018, 40203876. 40203876

[11] European Association of Urology Guidelines Panel on Testicular Cancer , EAU Guidelines on Testicular Cancer, Proceedings of the EAU Annual Congress London 2026, 2026, EAU Guidelines Office.

[12] Comiter C. V. , Renshaw A. A. , Benson C. B. , and Loughlin K. R. , Burned-Out Primary Testicular Cancer: Sonographic and Pathological Characteristics, Journal of Urology. (1996) 156, no. 1, 85–88, 10.1016/S0022-5347(01)65947-0, 8648846. 8648846

